# Effect of Immediately-After-Birth Weaning on the Development of Goat Kids Born to Small Ruminant Lentivirus-Positive Dams

**DOI:** 10.3390/ani9100822

**Published:** 2019-10-17

**Authors:** Tomasz Nalbert, Michał Czopowicz, Olga Szaluś-Jordanow, Agata Moroz, Marcin Mickiewicz, Lucjan Witkowski, Iwona Markowska-Daniel, Ryszard Puchała, Emilia Bagnicka, Jarosław Kaba

**Affiliations:** 1 Division of Veterinary Epidemiology and Economics, Institute of Veterinary Medicine, Warsaw University of Life Sciences, Nowoursynowska 159c, 02-776 Warsaw, Poland; tomasz_nalbert@sggw.pl (T.N.); agata_moroz@sggw.pl (A.M.); marcin_mickiewicz@sggw.pl (M.M.); lucjan_witkowski@sggw.pl (L.W.); iwona_markowska_daniel@sggw.pl (I.M.-D.); jaroslaw_kaba@sggw.pl (J.K.); 2 Department of Small Animal Diseases with Clinic, Faculty of Veterinary Medicine, Warsaw University of Life Sciences, Nowoursynowska 159c, 02-776 Warsaw, Poland; olga_szalus@sggw.pl; 3 Applied Physiology Unit, Military Institute of Hygiene and Epidemiology, Kozielska 4, 01-001 Warsaw, Poland; rapuchala@gmail.com; 4 Institute of Genetics and Animal Breeding, Polish Academy of Sciences, Postępu 36A, Jastrzębiec, 05-552 Magdalenka, Poland; e.bagnicka@ighz.pl

**Keywords:** body weight, caprine arthritis-encephalitis, mixed linear model, mortality, snatching, weaning

## Abstract

**Simple Summary:**

Caprine arthritis-encephalitis (CAE) is one of the most important and devastating viral diseases of small ruminants in developed countries. The disease spreads easily from one goat to another, and neither curative therapy nor preventive vaccinations exist to break the chain of infections. Hence, the only way to suppress the epidemic in a herd is to identify and cull infected goats. However, it is both costly and cruel as most of them remain apparently healthy for many months or even years, yet they constantly shed the virus. Therefore, many farmers decide to wean kids away from their infected mothers as soon as they are born and keep them in separation, feeding them on bovine or artificial colostrum and milk. Even though this approach is quite effective, it may be recommended only if it has no negative impact on kids’ development. Therefore, we conducted the study which compared the development of kids weaned immediately after birth and kids kept with mothers. We showed that early weaning did not seem to have any detrimental effect on kids’ health. On this basis, we conclude that this procedure may be an advisable alternative for those farmers who cannot afford to implement radical eradication program in their herds.

**Abstract:**

A longitudinal study was carried out to investigate the influence of two different rearing systems of young kids on their development to sexual maturity. Kids born to small ruminant lentiviruses-infected (SRLV) female goats were split into two groups: the immediately-after-birth weaned group and the unweaned group. Kids’ body weight (BWT) was measured before the first consumption of colostrum, and then at the age of one week, and one, two, four, and seven months. The relationship between the rearing system and BWT at each age was investigated using mixed linear models adjusted for potential confounders. The mean BWT of kids of the immediately-after-birth weaned group was significantly lower at the age of one week, one month, and two months, and then the difference became insignificant. The mean daily body weight gain (DWG) was significantly lower in the immediately-after-birth weaned group during the whole first month of life, but then DWG in both groups became equal. Crude mortality rate did not differ significantly between groups. This study shows that weaning kids immediately after birth does not appear to have any negative impact on kids’ development except transient growth retardation, which is fully compensated until they reach sexual maturity.

## 1. Introduction

Caprine arthritis-encephalitis (CAE) is an infectious and contagious disease caused by small ruminant lentiviruses (SRLV). The disease is widespread in various regions of the world, including Poland [1,2]. The most prominent clinical signs of CAE are arthritis and chronic wasting. They deteriorate animals’ condition and welfare, decreasing their productivity and necessitating culling of diseased goats [3]. However, clinical signs develop after several years of infection and only in less than a half of infected goats [4,5]. Effects of subclinical infection on productivity are not clear. Even if some studies show negative impact on milk and cheese yield, its magnitude is small [6,7]. Furthermore, infection of mothers does not seem to interfere with reproduction [8] and development of offspring [9]. All these observations raise doubts regarding the most optimal control measures of CAE. While it is undisputable that total eradication of SRLV from a population may only be achieved by culling of all infected individuals, costs of such test-and-cull programs may easily outweigh benefits attained by a farmer. Therefore, less radical approaches may occasionally turn out to be useful. Immediate weaning of kids and raising them on bovine colostrum and milk replacers, also referred to as snatching [10], is the most often recommended protocol. This approach has strong theoretical background as the lactogenic route (i.e. consumption of SRLV-laden colostrum and milk by a kid) plays basic role in CAE transmission [11,12]. Unfortunately, the infection may also spread through direct and indirect contact between adult goats [12]. As a consequence, such a control program is not capable of eradicating the disease, however it may considerably reduce the within-herd prevalence [13,14]. On the other hand, the influence of weaning of kids immediately after birth on their development and health is unknown as the typical age of weaning considered as an early weaning in previous studies regarding development of young goats has been three to four weeks [15,16]. Therefore, we carried out a longitudinal study to compare the influence of two different rearing systems of young kids on their growth rate and crude mortality rate until they reach sexual maturity.

## 2. Materials and Methods

### 2.1. Animals and Data Collection

The study was carried out in the research dairy goat herd of the Institute of Genetics and Animal Breeding of the Polish Academy of Sciences in 2014 and 2015. All adult goats (>six-months-old) in the herd had been regularly serologically tested for SRLV infection twice a year (in the spring and autumn) using commercial immunoenzymatic assay ID Screen MVV-CAEV Indirect Screening test (ID.vet Innovative Diagnostics, Grabels, France). Regular serological testing was a part of the voluntary CAE control program. Blood collection was approved by the third Local Ethical Committee in Warsaw, Poland (Approval No. 31/2013, 22 May 2013). Other procedures did not require ethical approval according to the Polish legal regulations [17].

In total, 70 goat kids born to 31 does were enrolled in the study, including 41 kids born to 20 different does in 2014, and 29 kids born to 15 different does in 2015, which amounted to 35 parturitions. Four does gave birth to study kids in both the study years. Does were between three and nine-years-old with the median of five years. They belonged to the Polish White Improved (PWI, n = 19) or Polish Fawn Improved breed (PFI, n = 12). Five does (16%) were SRLV-seronegative, and each of them was included in the study only in a single year so they accounted for five parturitions. The remaining 26 does (84%) were seropositive, and four of them were included in the study twice, so they accounted for 30 parturitions. The time which elapsed from seroconversion to parturition ranged from one to seven years (Supplementary Materials Table S1). In 35 parturitions, eight litters comprised singletons (23%), 18 – twins (51%), eight – triplets (23%) and one – quadruplets (3%). One kid born in a twin litter and one from a triple litter died soon after birth and were dropped from analysis. 45 kids (64%) were males (26 born in 2014), and 25 (36%) were females (15 born in 2014).

70 kids were split into two groups based on a different rearing system. 33 kids (47% of 70 kids, including 10 males) were weaned immediately after birth and kept in isolation from their mothers. For the first five days of life, kids were fed with bovine colostrum 150–250 mL, four times a day through a nipple bottle. Subsequently, they were switched onto the whey-based milk replacer (Sprayfo Primo Goat Kid, Trouw Nutrition, Poland – composition according to the manufacturer: lactose 36.5%, protein 22%, fat 22%, vitamins & minerals 8%, moisture 3%), which was served from troughs three times a day at daily doses increasing from 1 l to 2 l till the age of five weeks. This gradually tapered in next weeks, to be finally discontinued at the age of three months. Starting from the second week of life, fresh hay was available at will. From the third week of life, mixed concentrates based on crushed oat were gradually introduced at a daily dose increasing along with kids’ age from 100 to 300 g per an individual. This group was referred to as the immediately-after-birth weaned group.

The remaining 37 kids (53% of 70 kids; including 35 males) were urged to consume their dams’ colostrum immediately after birth. They were left with their mothers for next three weeks so that they could suckle at will. This group was referred to as the unweaned group. Then, they joined the immediately-after-birth weaned group and all feeding procedures were the same in both groups. At the age of three months, all 35 male kids belonging to the unweaned group were surgically castrated with pain relief using a non-steroid anti-inflammatory drug, meloxicam at the dose of 0.5 mg/kg s.c. administered once, an hour before the surgery (Metacam, Boehringer Ingelheim Vetmedica GmbH, Germany).

Kids were weighed before the first consumption of colostrum (birth body weight, BW), and then at the age of one week, one month, two months, four months, and seven months. A portable electronic animal plate weighing scale up to weighing precision of ± 0.05 kg (model SD75L, Ohaus Corporation, Parsippany, NJ, USA) was used and the body weight of each kid (BWT) was recorded in kg with one decimal place. Daily body weight gain (DWG) was calculated by subtracting BWT at the beginning of a given period from BWT at the end of this period and dividing by the number of days this period covered. Moreover, all mortalities were recorded. Kids were weighed twice in the first month (age of one week and one month) to see when the difference in body weight would emerge between groups. The age of seven months was a typical age of mating goats for the first time in this herd. Therefore, this moment was considered as sexual maturity and chosen for termination of the study.

### 2.2. Statistical Analysis

The age of goats was presented as the median and range. BWT and DWG were presented as the arithmetic mean and standard deviation (± SD), adjusted by confounders. At each age BWT was compared between the immediately-after-birth weaned and unweaned group using the mixed linear model (MLM) which resulted in five MLMs. The main explanatory variable ‘group’ was fitted as a fixed effect and included as a category of immediately-after-birth weaned kids (X_IW_) with the unweaned kids as a reference category to investigate the influence of this system of rearing on BWT. The variable “doe” (*D*) was forced into the model and fitted as a random effect to control for the dependence of measurements obtained from the various number of kids born by the same doe. Moreover, each MLM comprised two potential confounders fitted as fixed effects which were then eliminated based on their F statistics according to the backward stepwise procedure. These confounders were: the numerical variable “birth body weight” (X_BW_) to control for the initial body weight of a kid, and the categorical variable “kid’s sex” included as a category of males (X_males_) with females as a reference category to control for sexual dimorphism, and the fact that males significantly outnumbered females in the unweaned group at each age (*p* < 0.001). BW was mean centered by subtracting its mean from each individual score before entering into the model to let the intercept be the mean BWT when a kid was born with BW equal to the mean BW. 

The five models for each age were described by the following equation:

(1) $$Y_{\mathrm{BWT}}=B_{0}+\left(B_{\mathrm{BW}}\times X_{\mathrm{BW}}+B_{\mathrm{males}}\times X_{\mathrm{males}}\right)+B_{\mathrm{IW}}\times X_{\mathrm{IW}}+D+\varepsilon$$

Y_BWT_ stood for BWT at a given age estimated by the model. B_0_ was an intercept and B with a relevant subscript stood for the coefficient of regression of a given explanatory variable. Epsilon letter (ε) signified the residual (error). Parentheses indicated confounders which were retained in or removed from the model on the basis of the backward stepwise procedure. Two final models included both confounders (MLM at the age of one and four months), two other models included only BW (MLM at the age of one week and two months), and one model did not contain any of the two confounders (MLM at the age of seven months) (Supplementary Materials Table S2). SDs were calculated from the standard error (SE) obtained from MLM according to the formula:

(2) $$\mathrm{SD}=\mathrm{SE}\times \sqrt{n}$$

where n was the group size at a given age.

Another five MLMs were developed to investigate DWG in five subsequent periods of life of kids: from birth to the age of one week (seven days), from the age of one week to one month (23 days), one to two months (30 days), two to four months (60 days), and four to seven months (90 days). Likewise in the former MLMs the main explanatory variable “group” was fitted as a fixed effect and included as a category of the immediately-after-birth weaned kids (X_IW_) with the unweaned kids as a reference category. Two confounders were forced in the model: the variable “doe” (*D*) fitted as a random effect and “kid’s sex” fitted as a fixed effect and included as a category of males (X_males_) with females serving as a reference category. No stepwise procedure was used. 

The models were described by the following equation:

(3) $$Y_{\mathrm{DWG}}=B_{0}+B_{\mathrm{males}}\times X_{\mathrm{males}}+B_{\mathrm{IW}}\times X_{\mathrm{IW}}+D+\varepsilon$$

Categorical variables (i.e., kids’ sex and mortalities) were presented as the count and percentage in the group and compared between groups using the Pearson’s chi-square test or Fisher’s exact test if expected count in the contingency table was below five. Confidence intervals were calculated using the Wilson score method. All statistical tests were two-tailed. A significance level (α) was set at 0.05. Statistical analysis was performed in TIBCO Statistica 13.3.0 (TIBCO Software Inc., Palo Alto, CA, USA) and IBM SPSS Statistics 24 (IBM Corporation, Armonk, NY, USA).

## 3. Results

Up to the age of one month, all 70 kids were alive, and they were weighed three times (at birth, at the age of one week and one month). A week later, one male from the unweaned group died. Between the age of 10 and 14 weeks, five females from the immediately-after-birth weaned group and one male from the unweaned group died. At the age of six months, one male and one female from the immediately-after-birth weaned group were sold. Resultantly, the number of kids included in the analysis dropped during the study period by seven in the immediately-after-birth weaned group and by two in the unweaned group. There were five deaths in the immediately-after-birth weaned group (15% of 33 kids), and two deaths (5% of 37 kids) in the unweaned group. This resulted in the crude mortality rate of 15% (CI 95%: 7% to 31%) and 5% (CI 95%: 2% to 18%), respectively, and this difference was statistically insignificant (*p* = 0.175). However, the power of such a comparison at α = 0.05 and group sizes of 33 and 37 was only 29%.

**Figure 1:**
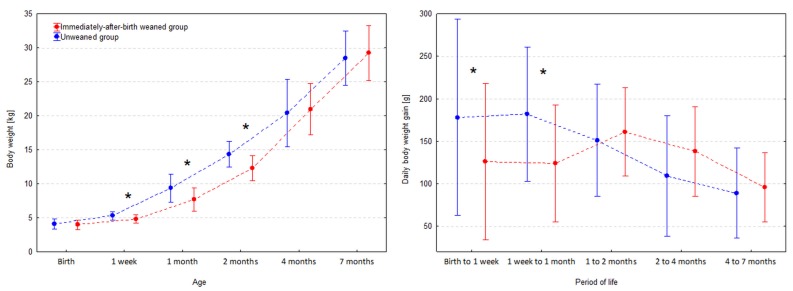
Body weight and daily body weight gain of kids (shown as means and SD) during the study period. The difference significant at α = 0.05 is indicated by an asterisk (*).

BW did not differ significantly between the two groups of kids (*p* = 0.323). Then, mean BWT of kids from the immediately-after-birth weaned group became significantly lower: by 0.5 kg (CI 95%: 0.2 to 0.7 kg; *p* = 0.001) at the age of one week, by 1.7 kg (CI 95%: 0.8 to 2.5 kg; *p* < 0.001) at the age of one month, and by 2.1 kg (CI 95%: 1.1 to 2.9 kg; *p* < 0.001) a month later. At the age of four months, the difference in the mean BWT became insignificant (*p* = 0.643) and remained so at the age of seven months (*p* = 0.472) (Figure 1, Table 1, Supplementary Materials Table S3).

BW significantly contributed to BWT of kids until they turned four-months-old. On the other hand, the independent role of kid’s sex was significant only at the age of one and four months when males were heavier than females by 0.9 kg (CI 95%: 0.1 to 1.7 kg, *p* = 0.025) and 2.5 kg (CI 95%: <0.1 to 4.9 kg, *p* = 0.048), respectively (Table S2, Table S3).

Controlling for an individual effect of a doe and kid’s sex mean DWG was significantly lower in the immediately-after-birth weaned group only during the first week of life (*p* = 0.036) and between the first week and first month of life (*p* = 0.001) – by 52 g (CI 95%: 4 to 101 g) and 59g (CI 95%: 27 to 90 g), respectively. Then, DWG was similar in both groups (Figure 1, Table 2, and Supplementary Materials Table S4). Between the age of two and four months kids from the immediately-after-birth weaned group tended to have higher mean DWG: by 29 g (CI 95%: −2 to 60 g). This difference was statistically insignificant (*p* = 0.069), however seemed to be responsible for equalization of BWT at the age of four months.

## 4. Discussion

Our study shows that weaning kids immediately after birth and rearing them on bovine colostrum and milk replacer results in transient growth retardation compared to kids suckling their mothers at will for the first three weeks of life. Growth retardation is already evident in one-week-old kids and persists till the age of two months. Over the next two months, kids weaned immediately after birth seem to grow faster to catch up with their counterparts from the unweaned group. At the age of four months, the difference in BWT vanishes completely and remains unapparent when goats enter the reproduction cycle. Moreover, weaning kids immediately after birth does not appear to increase mortality. However, the lack of influence of this system of rearing on crude mortality rate should be treated with caution due to the low power of this analysis.

BW proved to determine BWT of kids before maturity, and this relationship was significant at each age except seven months. Male sex was in general an insignificant determinant of BWT, even at the age of seven months when goats reach maturity and are usually bred. Weak influence of kids’ sex on BWT in premature goats may be explained by the leading role of BW as a BWT determinant, which means that male kids are born with higher BW and simply therefore they are heavier in consecutive months. At the age of seven months, BWT of males and females is similar which is consistent with previous observations [18].

Observation of DWG shows that kids grow faster only during this time when they stay with their mothers. One month after weaning, DWG becomes equal in both groups. This may indicate that consumption of mother’s milk is a key explanation of the faster growth. Previous studies in calves have shown that ad libidum feeding during the first weeks of life promotes growth [19,20] so significantly higher BWT of kids accompanying their mothers in the first weeks of life may simply result from the higher amount of feed consumed thanks to frequent chances to suckle during a day. However, in other studies [21,22], calves fed on the whole milk proved to grow faster than calves fed on milk replacer despite the fact that the amounts of both feeds consumed by calves were identical. Therefore, our study may substantiate the observation that mother’s milk provides the best nourishment, probably because of more optimal bioavailability of nutrients. As the milk replacer used in our study had balanced composition and high quality of components, mother’s milk may possibly be the source of some unknown growth-stimulating substances. Whether the results would be the same if bovine whole milk or heat-treated caprine whole milk was used instead of the milk replacer is yet to be verified.

On the other hand, nature of growth retardation caused by weaning immediately after birth is transient which is consistent with some observations made in dairy cattle [23,24]. Capability of fast compensation of retarded growth implies that weaning immediately after birth does not seem to hamper further performance of dairy goats. The difference in BWT disappeared between the age of two and four months, however the second month of life was also the moment when growth of kids of the unweaned group considerably slowed down (from 180 to 150g daily on average) while it accelerated in kids weaned immediately after birth (from 125 to 160g daily on average). Slower growth could be explained by stress caused by weaning. Weaning, especially abrupt with no further contact with dams allowed, has been shown to be a strong stimulator of stress reaction [25,26] with the release of high amounts of catabolic glucocorticosteroids [27] and disruption of the intestinal barrier [28]. Growth retardation has been observed to be more severe and longer the younger kids were at weaning - it lasted roughly three weeks when kids were weaned at the age of four weeks and two weeks when weaned a month later [29,30]. On the other hand, a compensatory overgrowth following the initial latent period has been observed and goats at the age of five months have exhibited similar weight gains regardless of the age at weaning [16,29,30,31]. Nevertheless, in most of studies the youngest kids weaned were three-weeks-old, and this age was referred to as early weaning. Our study shows that even when kids are weaned immediately after birth, growth retardation lasts no longer than a month. So, extremely early weaning simply shifts the moment when weaning-associated growth slump occurs but does not seem to make it more detrimental to kids than weaning at the age of three weeks.

Surgical castration of males was performed at the age of three month in only one group of kids (the unweaned group) since this study was a part of another scientific project regarding the pathogenesis of CAE. Kids kept with mothers were expected to get infected with SRLV via lactogenic route and to form a group of SRLV-infected goats in the future. Therefore, the unweaned group comprised mainly male kids, as female kids were simply more important for herd managers, so they were weaned immediately after birth to prevent lactogenic infection. As goats from the unweaned group (mainly males) were kept in the herd longer than only seven months described in this article and they were not intended for breeding, they were castrated to reduce aggression and nasty smell. However, castration of individuals from only one group is unlikely to have biased the results of our study, as surgical neutering has been shown not to have any short-term effect on the body weight gain of males, especially if performed with proper pain mitigation [32], just like it was done in our study.

Even though the does enrolled in the study belonged to two different breeds (PWI and PFI), we decided not to include their breed as a confounder in the analysis as no studies so far carried out have shown any real differences between the two breeds [33]. Those two breeds were developed by mating Saanen (PWI) or French Alpine goats (PFI) with many other local breeds, and intensive selection of purebreds has begun only recently [34]. Share of genes of those breeds in the offspring were neither known nor constant. Therefore, we believe that it was not justified to include the breed as a fixed effect and sufficient part of the role of breed in body weight variability was covered by a random effect of the mother.

## 5. Conclusions

In conclusion, our study shows that weaning of kids immediately after birth causes only transient growth retardation, which is fully compensated until goats reach sexual maturity. Therefore, this manner of weaning of kids can be safely used as a part of CAE control programs in those herds which cannot afford to implement the test-and-cull eradication program.

## Figures and Tables

**Table 1:** Body weight (BWT) of kids, shown as mixed linear model-estimated means and standard deviation (SD) during the study period.

Age	Immediately-after-Birth Weaned Group		Non-Weaned Group		Mean Difference in BWT (CI 95%) [kg]	MLM *p*-Value
	n (Male-to-Female Ratio)	Mean ± SD [kg]	n (Male-to-Female Ratio)	Mean ± SD [kg]		
Birth	33 (10:23)	3.95 ± 0.71	37 (35:2)	4.10 ± 0.74	–	0.323
1 week	33 (10:23)	4.80 ± 0.60	37 (35:2)	5.27 ± 0.63	−0.47 (−0.72, −0.21)	0.001
1 month	33 (10:23)	7.68 ± 1.73	37 (35:2)	9.35 ± 2.09	−1.67 (−2.52, −0.82)	<0.001
2 months	33 (10:23)	12.29 ± 1.85	36 (34:2)	14.35 ± 1.89	−2.06 (−2.94, −1.18)	<0.001
4 months	28 (10:18)	20.96 ± 3.79	35 (33:2)	20.42 ± 4.96	0.54 (−1.78, 2.85)	0.643
7 months	26 (9:17)	29.22 ± 4.00	35 (33:2)	28.47 ± 4.01	0.75 (−1.33, 2.82)	0.472

**Table 2:** Daily weight gain (DWG) of kids [g] shown as mixed linear model-estimated means and standard deviation (SD) during the study period.

Period of Time	Immediately-after-Birth Weaned Group	Unweaned Group	Mean Difference in DWG [g] (CI 95%)	MLM *p*-Value
	Mean ± SD [g]	Mean ± SD [g]		
Birth to 1 week of life	126 ± 92	178 ± 116	−52 (−101 to −4)	0.036
1 week to 1 month of life	124 ± 69	182 ± 79	−59 (−90 to −27)	0.001
1 to 2 months of life	161 ± 52	151 ± 66	10 (−20 to 39)	0.518
2 to 4 months of life	138 ± 53	109 ± 71	29 (−2 to 60)	0.069
4 to 7 months of life	96 ± 41	89 ± 53	6 (−21 to 34)	0.634

## Supplementary Materials

The following are available online at https://www.mdpi.com/2076-2615/9/10/822/s1, Table S1. Characteristics of 31 does and their 70 kids enrolled in the study, Table S2. Mixed linear models (MLM) investigating the effect of weaning of kids immediately after birth on their body weight (BWT) at subsequent steps of backward stepwise elimination procedure, Table S3. Mixed linear models investigating the effect of weaning of kids immediately after birth on their body weight (BWT) at various age, Table S4. Mixed linear models investigating the effect of weaning of kids immediately after birth on the daily body weight gain (DWG) in five periods of kids’ life.

## Abbreviations

BW	birth body weight of a kid
BWT	body weight of a kid
CAE	caprine arthritis-encephalitis
CI	confidence interval
DWG	daily body weight gain
MLM	mixed linear model
SD	standard deviation
SE	standard error
SRLV	small ruminant lentiviruses
