# Supplementary material for: Effect of Immediately-After-Birth Weaning on the Development of Goat Kids Born to Small Ruminant Lentivirus-Positive Dams
Source: Animals (Basel). 2019 Oct 17;9(10):822. doi: 10.3390/ani9100822 (PMC6827000; doi:10.3390/ani9100822)
Supplement: Supplementary file 1 [file animals-09-00822-s001.zip › Table S1.docx]

**Table S1**. Characteristics of 31 does and their 70 kids enrolled in the study

| **Doe** | **Breed** | **Year Born** | **SRLV Status** | **Year when Seroconverted** | **Kids Born in** | |
| --- | --- | --- | --- | --- | --- | --- |
|  |  |  |  |  | **2014** | **2015** |
| 1 | PWI | 2005 | positive | 2007 | m(1),m(0),m(0) | - |
| 2 | PWI | 2009 | positive | 2011 | m(0) | - |
| 3 | PWI | 2009 | positive | 2012 | m(0) | m(0) |
| 4 | PFI | 2009 | positive | 2014 | - | m(0),m(0) |
| 5 | PWI | 2010 | positive | 2011 | m(0),m(0) | - |
| 6 | PFI | 2010 | positive | 2013 | m(0),f(1) | - |
| 7 | PWI | 2006 | positive | 2013 | m(0),m(0),f(1) | - |
| 8 | PFI | 2006 | negative | - | - | f(1) |
| 9 | PWI | 2010 | positive | 2011 | f(1),f(1) | - |
| 10 | PFI | 2010 | positive | 2011 | f(1),f(1) | - |
| 11 | PWI | 2010 | negative | - | m(1),m(1),f(1) | - |
| 12 | PWI | 2011 | positive | 2013 | f(1),f(1) | - |
| 13 | PFI | 2011 | negative | - | - | m(1),f(1) |
| 14 | PWI | 2011 | positive | 2013 | m(0),f(1) | - |
| 15 | PWI | 2011 | positive | 2011 | m(0),f(1) | - |
| 16 | PWI | 2011 | positive | 2013 | m(0),f(0) | f(1),f(1) |
| 17 | PWI | 2012 | positive | 2014 | - | m(0),m(0) |
| 18 | PWI | 2012 | positive | 2014 | - | f(1) |
| 19 | PFI | 2012 | negative | - | - | m(0),m(0) |
| 20 | PWI | 2012 | positive | 2014 | - | m(1),m(1) |
| 21 | PFI | 2012 | positive | 2015 | - | m(1),f(1) |
| 22 | PWI | 2008 | positive | 2013 | m(0) | m(0),m(0),f(1) |
| 23 | PWI | 2007 | positive | 2010 | m(0),m(0),m(0) | - |
| 24 | PFI | 2008 | positive | 2010 | m(0) | - |
| 25 | PFI | 2008 | positive | 2013 | m(0),f(1),f(1) | - |
| 26 | PFI | 2008 | negative | - | - | m(1),f(1) |
| 27 | PFI | 2008 | positive | 2013 | m(0),f(0) | m(0),m(0) |
| 28 | PWI | 2008 | negative | - | m(1) | - |
| 29 | PWI | 2009 | positive | 2013 | m(1),m(0),m(0) | m(0),m(0), f(1),f(1) |
| 30 | PWI | 2009 | positive | 2013 | - | m(0) |

PWI – Polish White Improved, PFI – Polish Fawn Improved, m – male kid, f – female kid, (1) – early weaned, (0) – left with mothers
