# Supplementary material for: Effect of Immediately-After-Birth Weaning on the Development of Goat Kids Born to Small Ruminant Lentivirus-Positive Dams
Source: Animals (Basel). 2019 Oct 17;9(10):822. doi: 10.3390/ani9100822 (PMC6827000; doi:10.3390/ani9100822)
Supplement: Supplementary file 1 [file animals-09-00822-s001.zip › Table S2.docx]

**Table S2**. Mixed linear models (MLM) investigating the effect of weaning of kids immediately after birth on their body weight (BWT) at subsequent steps of backward stepwise elimination procedure. Variables in bold were included in the final model.

| **Variable** | **MLM – 1^st^ step**  **(initial model)** | **MLM – 2^nd^ step** | **MLM – 3^rd^ step** |
| --- | --- | --- | --- |
| Age of 1 week | | | |
| Weaning Immediately after birth | F_1,66_ = 4.14, *p* = 0.046 | F_1,67_ = 12.98, *p* = 0.001 | - |
| birth body weight of a Kid (BW) | F_1,65_ = 92.07, *p* < 0.001 | F_1,66_ = 98.65, *p* < 0.001 | - |
| Kid’s sex | F_1,60_ = 0.95, *p* = 0.334^a^ | - | - |
| Age of 1 month | | | |
| Weaning immediately after birth | F_1,62_ = 15.48, *p* < 0.001 | - | - |
| Birth body weight of a kid (BW) | F_1,65_ = 31.44, *p* < 0.001 | - | - |
| Kid’s sex | F_1,55_ = 5.28, *p* = 0.025 | - | - |
| Age of 2 months | | | |
| Weaning immediately after birth | F_1,60_ = 5.92, *p* = 0.018 | F_1,60_ = 21.91, *p* < 0.001 | - |
| Birth body weight of a kid (BW) | F_1,46_ = 11.86, *p* = 0.001 | F_1,45_ = 13.16, *p* = 0.001 | - |
| Kid’s sex | F_1,65_ = 2.93, *p* = 0.092^a^ | - | - |
| Age of 4 months | | | |
| Weaning immediately after birth | F_1,54_ = 0.22, *p* = 0.643 | - | - |
| Birth body weight of a kid (BW) | F_1,41_ = 9.00, *p* = 0.005 | - | - |
| Kid’s sex | F_1,59_ = 4.09, *p* = 0.048 | - | - |
| Age of 7 months | | | |
| Weaning immediately after birth | F_1,48_ = 0.19, *p* = 0.668 | F_1,50_ = 0.84, *p* = 0.365 | F_1,59_ = 0.52, *p* = 0.472 |
| Birth body weight of a kid (BW) | F_1,27_ = 1.69, *p* = 0.205 | F_1,26_ = 1.62, *p* = 0.215 | - |
| Kid’s sex | F_1,56_ = 0.18, *p* = 0.678^a^ | - | - |

^a^ variables eliminated from MLM at subsequent steps
