# Supplementary material for: Effect of Immediately-After-Birth Weaning on the Development of Goat Kids Born to Small Ruminant Lentivirus-Positive Dams
Source: Animals (Basel). 2019 Oct 17;9(10):822. doi: 10.3390/ani9100822 (PMC6827000; doi:10.3390/ani9100822)
Supplement: Supplementary file 1 [file animals-09-00822-s001.zip › Table S3.docx]

**Table S3**. Mixed linear models (MLM) investigating the effect of weaning of kids immediately after birth on their body weight (BWT) at various age

| **Variable** | **BWT at the Age of 1 Week** | | | **BWT at the Age of 1 Month** | | | **BWT at the age of 2 Months** | | | **BWT at the Age of 4 Months** | | | **BWT at the Age of 7 Months** | | |
| --- | --- | --- | --- | --- | --- | --- | --- | --- | --- | --- | --- | --- | --- | --- | --- |
|  | **Estimate of the Model ^a^** | **Test Statistic** | ***p*-Value** | **Estimate of the Model ^a^** | **Test Statistic** | ***p*-Value** | **Estimate of the Model ^a^** | **Test Statistic** | ***p*-Value** | **Estimate of the Model ^a^** | **Test Statistic** | ***p*-Value** | **Estimate of the Model ^a^** | **Test Statistic** | ***p*-Value** |
| Intercept | 5.32 ± 0.10 | - | - | 8.96 ± 0.49 | - | - | 14.42 ± 0.31 | - | - | 19.33 ± 1.32 | - | - | 28.47 ± 0.68 | - | - |
| Variables fitted as fixed effects | | | | | | | | | | | | | | | |
| Rearing system: |  |  |  |  |  |  |  |  |  |  |  |  |  |  |  |
| Non-weaned^b^ | 0 | - | - | 0 | - | - | 0 | - | - | 0 | - | - | 0 | - | - |
| Weaned immediately after birth | **−0.47 ± 0.13**  **(−0.72, −0.21)*** | -3.60 | 0.001 | **−1.67 ± 0.43**  **(−2.52, −0.82)*** | -3.93 | <0.001 | **−2.06 ± 0.44**  **(−2.94, −1.18)*** | -4.68 | <0.001 | 0.54 ± 1.16  (**−**1.78, 2.85) | 0.47 | 0.643 | 0.75 ± 1.04  (**−**1.33, 2.82) | 0.72 | 0.472 |
| Birth body weight | 1.05 ± 0.11  (0.84, 1.26)* | 9.93 | <0.001 | 1.51 ± 0.27  (0.98, 2.05)* | 5.61 | <0.001 | 1.26 ± 0.35  (0.56, 1.95)* | 3.63 | 0.001 | 2.12 ± 0.71  (0.69, 3.54)* | 3.00 | 0.005 | X | X | X |
| Kid’s sex |  |  |  |  |  |  |  |  |  |  |  |  |  |  |  |
| female^b^ | 0 | - | - | 0 | - | - | 0 | - | - | 0 | - | - | 0 | - | - |
| male | X^c^ | X | X | 0.93 ± 0.41 (0.12, 1.74)* | 2.30 | 0.025 | X | X | X | 2.45 ± 1.21  (0.03, 4.87)* | 2.02 | 0.048 | X | X | X |
| Variables fitted as random effects | | | | | | | | | | | | | | | |
| Doe | 0.12 ± 0.06 | 1.94 | 0.052 | 1.26 ± 0.46 | 2.70 | 0.007 | 0.27 ± 0.55 | 0.50 | 0.620 | 1.82 ± 2.78 | 0.65 | 0.513 | X | X | X |
| Residual | 0.18 ± 0.04 | 4.32 | <0.001 | 0.93 ± 0.22 | 4.33 | <0.001 | 2.90 ± 0.68 | 4.29 | <0.001 | 10.40 ± 2.82 | 3.69 | <0.001 | 16.04 ± 2.95 | 5.43 | <0.001 |

^a^ regression coefficient (±SE, and CI 95%) for variables fitted as fixed effects and variance (±SE) for variables fitted as random effects; ^b^ reference category; ^c^ factor eliminated in earlier steps of stepwise backward elimination procedure; * significant at α=0.05
