# Supplementary material for: Effect of Immediately-After-Birth Weaning on the Development of Goat Kids Born to Small Ruminant Lentivirus-Positive Dams
Source: Animals (Basel). 2019 Oct 17;9(10):822. doi: 10.3390/ani9100822 (PMC6827000; doi:10.3390/ani9100822)
Supplement: Supplementary file 1 [file animals-09-00822-s001.zip › Table S4.docx]

**Table S4**. Mixed linear models (MLM) investigating the effect of weaning of kids immediately after birth on the daily body weight gain (DWG) in 5 periods of kids’ life

| **Variable** | **DWG in the 1^st^ Week of Life** | | | **DWG between the Age of 1 Week and 1 Month** | | | **DWG between the Age of 1 and 2 Months** | | | **DWG between the Age of 2 and 4 Months** | | | **DWG between the Age of 4 and 7 Months** | | |
| --- | --- | --- | --- | --- | --- | --- | --- | --- | --- | --- | --- | --- | --- | --- | --- |
|  | **Estimate of the Model ^a^** | **Test Statistic** | ***p*-Value** | **Estimate of the Model ^a^** | **Test Statistic** | ***p*-Value** | **Estimate of the Model ^a^** | **Test Statistic** | ***p*-Value** | **Estimate of the Model ^a^** | **Test Statistic** | ***p*-Value** | **Estimate of the Model ^a^** | **Test Statistic** | ***p*-Value** |
| Intercept | 0.167 ± 0.027 | - | - | 0.163 ± 0.018 | - | - | 0.148 ± 0.016 | - | - | 0.095 ± 0.018 | - | - | 0.103 ± 0.015 | - | - |
| Variables fitted as fixed effects | | | | | | | | | | | | | | | |
| Raising system: |  |  |  |  |  |  |  |  |  |  |  |  |  |  |  |
| Non-weaned^b^ | 0 | - | - | 0 | - | - | 0 | - | - | 0 | - | - | 0 | - | - |
| Weaned immediately after birth | **−0.052 ± 0.024 (−0.101, -0.004)*** | -2.14 | 0.036 | **−0.058 ± 0.016 (−0.090, -0.027)*** | -3.67 | 0.001 | 0.010 ± 0.015 (**−**0.020, 0.039)* | 0.65 | 0.518 | 0.029 ± 0.016 (**−**0.002, 0.060) | 1.85 | 0.069 | 0.007 ± 0.014 (**−**0.021, 0.034) | 0.479 | 0.634 |
| Kid’s sex |  |  |  |  |  |  |  |  |  |  |  |  |  |  |  |
| female^b^ | 0 | - | - | 0 | - | - | 0 | - | - | 0 | - | - | 0 | - | - |
| male | 0.022 ± 0.024 (**−**0.025, 0.069) | 0.95 | 0.345 | 0.039 ± 0.015 (0.009, 0.069)* | 2.64 | 0.011 | 0.006 ± 0.015 (**−**0.024, 0.035) | 0.39 | 0.698 | 0.029 ± 0.016 (**−**0.002, 0.061) | 1.86 | 0.069 | **−**0.028 ± 0.015 (**−**0.057, 0.001) | **−**1.91 | 0.061 |
| Variables fitted as random effects | | | | | | | | | | | | | | | |
| Doe | 0.003 ± 0.001 | 1.96 | 0.050 | 0.002 ± 0.001 | 2.83 | 0.005 | 0.001 ± 0.001 | 1.38 | 0.168 | 0.001 ± 0.001 | 1.03 | 0.305 | X | X | X |
| Residual | 0.004 ± 0.001 | 4.24 | <0.001 | 0.001 ± 0.001 | 4.33 | <0.001 | 0.002 ± 0.001 | 5.02 | <0.001 | 0.002 ± 0.001 | 3.67 | <0.001 | 0.002 ± 0.001 | 5.39 | <0.001 |

^a^ regression coefficient (±SE, and CI 95%) for variables fitted as fixed effects and variance (±SE) for variables fitted as random effects; ^b^ reference category; * significant at α=0.05
