# Supplementary figures and images for: Effect of Immediately-After-Birth Weaning on the Development of Goat Kids Born to Small Ruminant Lentivirus-Positive Dams
Source: Animals (Basel). 2019 Oct 17;9(10):822. doi: 10.3390/ani9100822 (PMC6827000; doi:10.3390/ani9100822)

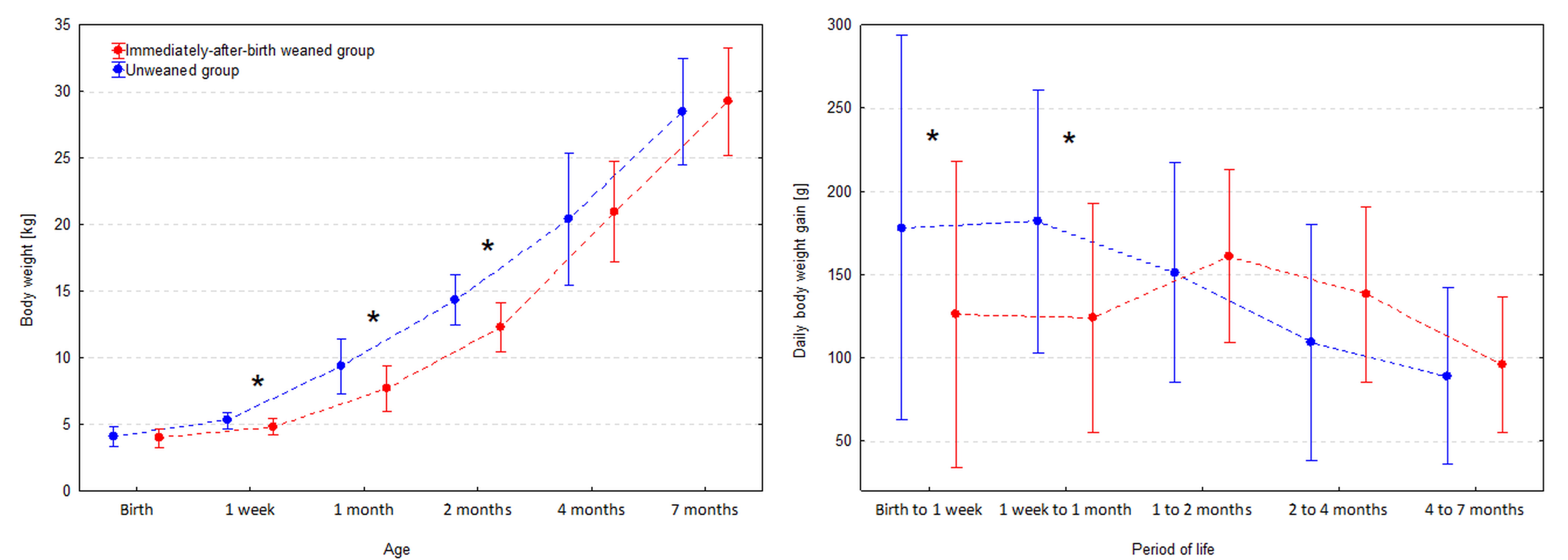

Supplement: Supplementary file 1 [file animals-09-00822-s001.zip › animals-593557-revised 2.jpg]
